# Dissecting the transactivation domain (tAD) of the transcription factor c‐Myb to assess recent models of tAD function

**DOI:** 10.1002/2211-5463.12978

**Published:** 2020-09-25

**Authors:** Guro Næs, Jan Ove Storesund, Priyanga‐Dina Udayakumar, Marit Ledsaak, Odd Stokke Gabrielsen

**Affiliations:** ^1^ Department of Biosciences University of Oslo Norway

**Keywords:** chromatin, c‐Myb, transactivation domain, transcription factor, yeast

## Abstract

Transcription factors use a DNA‐binding domain to localize their action and a transactivation domain (tAD) to stimulate activation of the associated gene. Recent work has renewed interest in how tADs activate genes, which remains poorly understood. Key features in the new models are exposure of short linear motifs (SLMs) and liquid–liquid phase separation (LLPS). Inspired by the new models for tAD function, we decided to revisit the tAD of the haematopoietic transcription factor c‐Myb by performing a mutational analysis to see how these new models fit and potentially explain the tAD behaviour of this master regulator. We know that c‐Myb has an acidic tAD, which contains a well‐characterized SLM in the form of a LxxLL motif. By testing 12 alanine‐scanning mutants and three mutants with major reorganization of its tAD in two mammalian reporter systems, we found a pattern of effects very close to what would be expected from the SLM‐exposure model, with strong effects exerted by both acidic replacements and SLM mutation. When the same mutants were tested in a yeast system, the pattern of effects was dramatically different, with the SLM mutation exerting no effect, and tAD behaviour was much less affected by small alterations, as would be expected from a LLPS model. These observations are discussed in the light of the two new tAD models, and a two‐step hypothesis for transactivation, combining both models, is proposed.

## Graphical abstract

Genes are activated by transcription factors through their transactivation domains (tADs); disordered regions that have been poorly understood. New models for tADs have recently been proposed. Here, these models were assessed through a mutational analysis of the tAD of the master regulator c‐Myb. Analysis in mammalian cells strongly supported the ‘SLM‐exposure model’. In yeast, tAD mutants behaved quite differently.

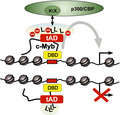

**Abbreviations**
2KR: double mutant of c‐Myb (K503R+K527R)
CBP: CREB binding protein
c‐Myb: v‐myb avian myeloblastosis viral oncogene homolog
CRD: C‐terminal regulatory domain
DBD: DNA‐binding domain
hcM: human c‐Myb
HKMT: histone lysine methyltransferase
IDR: intrinsically disordered region
KIX: kinase‐inducible domain (KID) interacting domain
LLPS: liquid–liquid phase separation
MLL: myeloid/lymphoid or mixed‐lineage leukaemia
p300: E1A binding protein p300 or histone acetyltransferase p300
SIM: SUMO‐interaction motifs
SLM: short linear motif
SUMO: small ubiquitin‐related modifier
tAD: transactivation domain
TF: transcription factor

Control of eukaryotic transcription is to a large extent dependent on transcription factors binding to regulatory elements in the genome and influencing the activity of the linked promoters. A transcription factor consists, as a minimum, of a DNA‐binding domain (DBD) and a transactivation domain (tAD). While DBDs in general are structured and functionally well understood [1, 2], the tADs are usually without a defined 3D structure, consisting of intrinsically disordered regions (IDRs) [3, 4]. This has limited our understanding of the mechanism of action of tADs, beyond their general function of recruiting coactivators and helping target genes to be turned on and activated. It is remarkable that such a key function as the one exerted by tADs is so poorly understood. However, this situation is about to change due to several seminal publications recently [5, 6].

Traditionally, tADs have been classified simply based on their amino acid composition, reflecting our limited understanding of tADs function. Initial studies classified tADs as acidic domains, enriched in D and E amino acid residues, and proposed a model where tADs were forming ‘acid blobs’ or ‘negative noodles’ [3, 7]. These were assumed to be able to form complexes with some essential component of the general transcriptional apparatus. Key activators studied were the yeast transcription factors Gal4p and Gcn4p. Gill and Ptashne [8] reported a mutational study on the Gal4p tAD, which showed a positive correlation between the strength of activation and the number of negative charges. The acidic tAD of the herpes simplex virus protein VP16 was shown to activate transcription remarkably efficiently in mammalian cells, suggesting a conserved mechanism of action [9]. We call this first model the ‘acidic blob model’.

The ‘acidic blob’ model was later extended to accommodate other specific amino acid residues, seeing tADs as domains enriched in glutamine (Q‐rich), proline (P‐rich) or isoleucine (Ile‐rich). The human transcription factor Sp1, binding to GC‐rich sequence elements, utilizes a Q‐rich tAD to activate promoters [8, 10]. Proline‐rich domains were identified in the human CTF/NF‐1 factor where the C‐terminal region includes an unusual type of tAD containing around 25% proline residues [10]. The *Drosophila* transcription factor NTF‐1, regulating several developmental genes, was found to have a single tAD with a high percentage of isoleucines. Changing as few as two of the isoleucines to alanine caused its activity to be significantly disrupted [11]. We call these hypothesis variants of a second model the ‘amino acid enrichment model’.

As more systematic studies of tADs accumulated, several examples revealed a higher importance of hydrophobic residues than acidic ones, even in acidic tADs [12, 13, 14, 15]. This led gradually to a third model where tADs were regarded as domains containing short linear motifs (SLMs), and hydrophobic and conserved sequence‐specific motifs mediating molecular interactions and being involved in recruitment of cofactors. Gcn4p is a typical example, containing a well‐characterized hydrophobic SLM, WxxLF, which is key to its activity [15, 16, 17]. Interestingly, the tAD of Gcn4p binds to its target, the mediator subunit Gal11/Med15, through a ‘fuzzy’ complex, allowing its SLM to bind in multiple different orientations when inserted into a hydrophobic cleft in Gal11p [16, 17]. This flexibility may explain how different tADs can bind to a few coactivators. We call this third hypothesis for the ‘SLM model’.

Recently, Staller *et al*. [5] proposed an interesting twist to these models, which could explain both the importance of hydrophobic residues and why tADs often are acidic. Their tAD model comprises a specific hydrophobic SLM embedded in a disordered acidic region. The intrinsic disorder and the acidic residues help keeping the hydrophobic motifs exposed to solvent, providing exposure to binding partners, thus facilitating coactivator interaction. This SLM‐exposure model was derived from a large‐scale rational mutagenesis scheme on Gcn4p that used a deconvolution strategy to dissect the contribution to activation of four tAD sequence features, namely acidity, hydrophobicity, SLMs and intrinsically disorder regions. Thus, the activity of thousands of variants was quantified in vivo in a yeast reporter system. The most‐active variants appeared to keep their aromatic residues exposed to the solvent. We call this fourth variant the ‘SLM‐exposure model’.

Another recent seminal contribution to our understanding of tAD structure and function has been the analysis of its phase‐separation properties. Liquid–liquid phase separation (LLPS) is a fundamental biophysical mechanism for organizing the intracellular space leading to the formation of membrane‐less condensates, which adopt round morphologies and coalesce into larger droplets upon contact with one another [18, 19]. Multiple weak interactions between IDRs are important driving forces for LLPS. Recently, several elements of the transcriptional apparatus have been reported to operate through LLPS‐related mechanisms, including the RNA polymerase II CTD [20, 21, 22], superenhancers [23], the mediator [24], elongation factors [25] and the chromatin template [26]. Similar studies of tAD function have led to a new model where tADs are functioning by forming phase‐separated condensates with the mediator to activate gene expression [4, 6]. Boija *et al*. [6] studied the tAD of three transcription factors, OCT4, GCN4 and the estrogen receptor, and found that they all interacted with the mediator and activated genes by the capacity of their tADs to form phase‐separated condensates. In addition, they found that the tAD amino acid residues required for phase separation with the mediator were also required for gene activation in vivo. We call this fifth hypothesis for the ‘LLPS model’.

We have for a long time been focusing on molecular mechanisms of c‐Myb [27, 28, 29, 30, 31]. The transcription factor c‐Myb is a pioneer factor involved in early differentiation and proliferation of haematopoietic cells, where it operates as a master regulator of self‐renewal and multilineage differentiation [31, 32, 33, 34, 35].

Several proteins have been reported to interact with c‐Myb and are crucial for its role as a transcriptional regulator. These include some transcription factors, such as C/EBPβ [36], and coactivators, such as p300/CBP and the HKMT complex MLL [37, 38]. p300/CBP are among the best‐characterized c‐Myb interaction partners. They stimulate c‐Myb‐dependent transcriptional activation by acetylating the C‐terminal regulatory domain (CRD) of c‐Myb and the neighbouring chromatin [37]. The main interaction surface has been mapped to the tAD of c‐Myb and the KIX domain of the coactivators [39]. The C/H2 domain of CBP may further enhance the interaction [40]. Our laboratory has identified several additional interaction partners of c‐Myb. The chromatin remodeller CHD3 was shown to interact with the DBD of c‐Myb and to function as a coactivator of c‐Myb activity [41]. We also found that c‐Myb cooperates with FLASH/CASP8AP2 in foci associated with active RNA polymerase II, leading to enhancement of Myb‐dependent gene activation [29]. Like CHD3, FLASH/CASP8AP2 was found to interact with the DBD of c‐Myb. Furthermore, two enzymes linking c‐Myb to the SUMO system were identified as interaction partners, namely the SUMO conjugation enzyme Ubc9 and the SUMO ligase PIAS1 [30, 42, 43].

Given the recent developments with new models for tAD function, we decided to revisit the tAD of c‐Myb and to perform a mutational analysis to see how these new models fit and explain the tAD behaviour of c‐Myb. We know that c‐Myb has an acidic tAD containing a well‐characterized SLM in the form of a LxxLL motif [39, 44, 45, 46, 47] and thus should have an organization relevant to the models listed above. By testing a series of alanine‐scanning mutants and mutants with major tAD reorganization in two mammalian reporter systems, we observed strong effects of both acidic replacements and SLM mutation, consistent with the SLM‐exposure model of Staller *et al*. [5]. When the same mutants were tested in a yeast system, the pattern was dramatically different with a tAD behaviour much less affected by small alterations.

## Materials and methods

### Cell culture, transfection and luciferase assays

Two cell lines were used: CV‐1 (ATCC® CCL‐70™ *Cercopithecus aethiops* Kidney Normal); and HEK293‐c1 [48], a derivative of HEK‐293 (ATCC® CRL‐1573™ *Homo sapiens* embryonic kidney). The cells were grown and transiently transfected with the indicated plasmids as previously described [28, 49]. Reporter assays in transiently transfected CV1 and HEK293‐c1 cells, stably transfected with a 5×Gal4‐luciferase reporter [48], were performed in triplicate (24‐well trays, 2 × 10^4^ CV‐1 cells/well or 3.4 × 10^5^ HEK 293‐c1 cells/well) using Luciferase Assay Reagent (Promega, Madison, WI, USA), each triplicate repeated in three independent experiments. Equal expression was confirmed by western blotting.

### Plasmid constructs

#### Mutant tAD fragments

The different tAD mutants were introduced in the pBS[Bgl]‐hcM‐EcoBgl plasmid by using QuikChange site‐directed mutagenesis [42]. The pBS[Bgl]‐hcM‐EcoBgl plasmid contains an internal segment of the human c‐Myb cDNA between the EcoRI and BglII site. The mutagenic oligos all introduced a silent diagnostic restriction site and had the following sequences (only upper strand oligo shown):

**Table:** 

Oligo name	Oligo sequence
TAD‐mut‐silent NcoI‐U	ACCATTGCCGACCACACCAGACCcCATGGAGACAGTGCACCTGTTTCC
TAD‐mut‐silent SacII‐U	GTAAATATAGTCAATGTCCCTCAGCCcGCgGCtGCAGCCATTCAGAGACACTATAATG
TAD‐mut3 DED‐U	GCCATTCAGAGACACTATAATGcTGcAGcCCCTGAGAAGGAAAAGCGAAT
TAD‐mut4 EKE‐U	GACACTATAATGATGAAGACCCaGccAAGGcgAAGCGAATAAAGGAATTAGAATTGC
TAD‐mut5 ELE‐U	GAGAAGGAAAAGCGAATAAAaGctTTAGcATTGCTCCTAATGTCAACCGA
TAD‐mut6 ENE‐U	GAATTGCTCCTAATGTCAACCGcGAAcGcGtTAAAAGGACAGCAGGTGCT
TAD‐mut7 KEKRIK‐U	TATAATGATGAAGACCCTGAGgctGAggcGgcAATAgcGGAATTAGAATTGCTCCTAATG
TAD‐mut8 RHY‐U	GCTGCCGCAGCCATTCAGgcAgcgTAcAATGATGAAGACCCTGAGAAGGA
TAD‐mut9 LxxLL‐U	AAGGAAAAGCGAATAAAGGAAgcAGAgctagcCgcAATGTCAACCGAGAATGAGC
TAD‐mut10 Y284A‐U	GCCGCAGCCATTCAGAGgCAtgcTAATGATGAAGACCCTGAGAAG
TAD‐mut11 VL‐U	AATGAGCTAAAAGGACAGCAGGcGgcgCCAACACAGAACCACACATGC
TAD‐mut12 WHSTT‐U	ACATGCAGCTACCCCGGGgcGgctAGCgCCgCCATTGCCGACCACACCAG
mini‐TAD‐U	gGAATTAGAgcTcCTCCTAATGccc
mini‐TAD‐L	catggggCATTAGGAGgAgcTCTAATTCcgc
deltaTAD‐U	gGAAgcAGAgctagcCgcAATGccc
deltaTAD‐L	catggggCATTgcGgctagcTCTgcTTCcgc

All positive clones were confirmed by restriction digestion, and the final construct was sequenced.

We used the same strategy to construct a variant of pBS[Bgl]‐hcM‐EcoBgl with two silent mutations, one on each side of the tAD region, creating unique restriction sites (NcoI and SacII), thus allowing simple replacement of the wild‐type tAD with synthesized oligo designs. The oligos ‘mini‐TAD‐L’ and ‘mini‐TAD‐U’ or ‘∆‐TAD‐L’ and ‘∆‐TAD‐U’ were annealed and inserted between the NcoI and SacII sites in the modified pBS[Bgl]‐hcM‐EcoBgl. In the same way, we inserted a fragment encoding a shuffled tAD made by gene synthesis (Eurofins Genomics, Ebersberg, Germany, pEX‐A128‐shuffled TAD) between the NcoI and SacII sites. All positive clones were confirmed by restriction digestion, and the final construct was sequenced.

#### Mammalian expression and reporter plasmids

The mutant tAD fragments were transferred to the mammalian expression vector pCIneoB‐hcM‐HA‐2KR (encoding a sumoylation‐deficient c‐Myb due to the double mutant K503R+K527R), which is a version of pCIneo‐hcM‐HA‐2KR [28] in which a BglII site in the vector was removed. The mutant tAD fragments were also transferred to the plasmid pCIneoB‐GBD2‐hcM‐2KR[194–640], which is a version of pCIneo‐GBD2‐hcM‐2KR[194–640] [28] in which a BglII site in the vector was removed. This plasmid encodes c‐Myb (2KR version) lacking its own DBD in fusion with the yeast Gal4p DBD DNA‐binding domain. The reporter plasmids used, pGL4b‐3xMRE(GG)‐myc, is previously described [28].

#### Yeast expression and reporter plasmids

The mutant tAD fragments were transferred to pYES2‐hcM, a yeast expression vector pYES2 (Thermo Fisher Scientific, Waltham, MA, USA) into which a full‐length cDNA encoding human c‐Myb was inserted between its KpnI+NotI sites. The pYES2‐hcM contains the *URA3* gene for selection in yeast and 2µ origin for high‐copy maintenance. Flanking the MCS is an inducible pGAL1 promotor and the *CYC1* transcription terminator. The reporter plasmid was a derivative of pYHLfus‐3xGG [50], where a fusion of *HIS3* and *lacZ* is driven by a synthetic Myb‐responsive promoter. The gene cassette with the dual‐reporter and its Myb‐responsive promoter was amplified by PCR using PfuUltra™ (Agilent, Santa Clara, CA, USA) and primers where a second BsrGI site was introduced through one of the primers. The purified PCR product was digested with BsrGI and inserted in pGADT7 (Clontech, Takara Bio Europe SAS, Saint‐Germain‐en‐Laye, France) digested with the same enzyme, resulting in the reporter pGADT7‐HLfus‐3xGG, where the selection marker is LEU2.

### Western analysis

For immunoblot detection, the following antibodies were used: rabbit anti‐c‐Myb H141 (sc7874; Santa Cruz Biotechnology, Dallas, TX, USA), mouse anti‐GAPDH (AM4300; Invitrogen, Thermo Fisher Scientific), IRDye800 anti‐rabbit (926‐32213; LI‐COR Biosciences, Lincoln, NE, USA) and IRDye680 anti‐mouse (926‐68072; LI‐COR Biosciences).

### Reporter assays in yeast

The yeast strain INVSC1 (Invitrogen, Thermo Fisher Scientific, genotype: *MATα*, *his3‐Δ1*, *leu2*, *trp1‐289*, *ura3‐52*) was transformed with both effector and reporter plasmids as previously described [50]. Yeast were grown in complete (YPD) or synthetic (SC) media [2% (w/v) glucose, 0.17% (w/v) nitrogen base and 0.5% (w/v) ammonium sulphate] supplemented with amino acids as described [51]. Transformants were selected on plates with medium selective for the plasmids.

Transactivation assays measuring β‐galactosidase (β‐Gal) activity in permeabilized cells were performed as previously described [50] with yeast INVSC1 transformed with both effector and reporter plasmids in either glucose medium (repressive state for *GAL1‐*controlled genes) or galactose medium (activating state for *GAL1‐*controlled genes). Transactivation assays, measuring growth ability of the transformants, were done on plates with galactose medium selective for the plasmids and lacking histidine.

## Results

### Analysis of two classical tAD models using c‐Myb variants with major reorganization of the tAD sequence

**Fig. 1:**
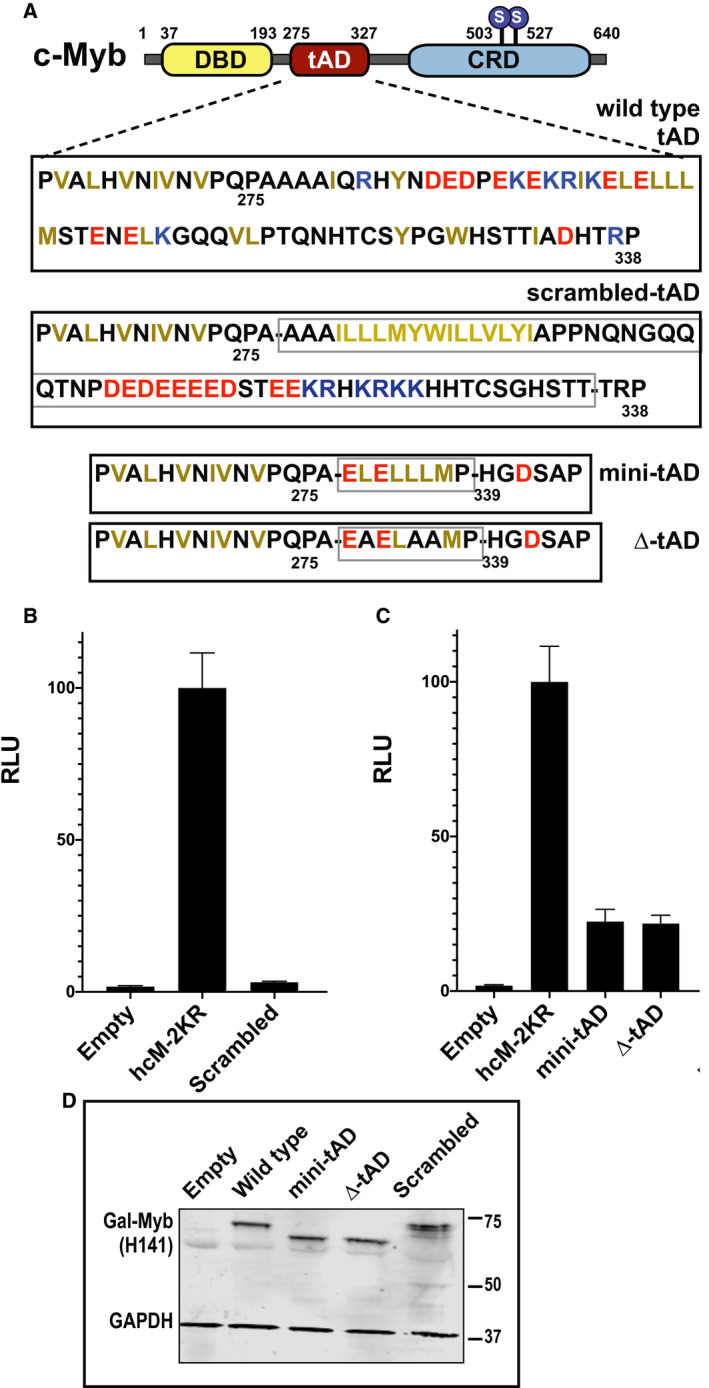
Analysis of synthetic variants of the tAD of c‐Myb. (A) Schematic presentation of human c‐Myb with its three main domains: DBD, DNA‐binding domain; tAD, transactivation domain; and CRD, C‐terminal regulatory domain. SUMO conjugation sites are indicated (S). The amino acid sequence of its wild‐type tAD and the synthetic variants analysed are shown. (B, C) The indicated synthetic tAD variants were analysed with the transfected reporter in CV‐1 cells. The results are presented as relative luciferase units (RLU) with wild‐type set to 100. The results represent the mean RLU ± SD of at least three independent assays performed in triplicates. (D) The western blots were analysed using anti‐c‐Myb (H141) and anti‐GAPDH primary antibodies.

For human c‐Myb, the exact borders of its tAD have not been defined, but based on early studies where a series of deletions were analysed, the tAD in c‐Myb was localized to be in the amino acid residue range of 275–327 [52]. Since we have previously reported strong effects on c‐Myb transactivation of mutants in the region 267–270 [53], we chose to analyse a slightly broader region in this work, 267–331 (Fig. 1A).

Before we performed a detailed analysis of the tAD of c‐Myb, we addressed two of the simple models for tAD function, the acidic blob model and the SLM model. The tAD of c‐Myb is acidic and contains an LxxLL motif that is involved in the interaction with the KIX domain of CBP and p300 [39, 44, 45, 46, 47]. We therefore asked two simple questions: Is the acidic property sufficient to generate a tAD in c‐Myb, and likewise, is the LxxLL motif sufficient for this function? To answer the first question, we asked how important the precise ordering of the amino acid residues is versus just the overall composition of the domain. Therefore, we tested a scrambled tAD with the same number of acidic residues and the same number and type of all other amino acids as the wild‐type. First, we introduced two silent restriction sites flanking the tAD and then inserted a synthetic duplex oligo between these two sites. Figure 1A shows the composition of the scrambled tAD versus the wild‐type sequence and the reporter activation results. The scrambled tAD was close to background activity despite having a long stretch of 8 + 2 acidic residues (Fig. 1B). Western analysis showed a similar steady‐state level of the scrambled tAD (Fig. 1D). Clearly, having the same overall composition as the original tAD is not sufficient to form an active tAD.

We also asked whether the LxxLL motif would be sufficient by itself to create a functional tAD in line with the SLM model. We used the same design as for the scrambled tAD to design a ‘mini‐tAD’, replacing the 77 residue tAD with a short 8 residue long sequence including the LxxLL motif (Fig. 1A). We similarly made a ‘∆‐tAD’ version as a negative control of the latter where the leucines in the LxxLL motif of the ‘mini‐tAD’ were replaced with alanines (Fig. 1A). Despite a strong drop in reporter activation, a small residual activity was observed (Fig. 1C). However, this was not dependent on the LxxLL motif since the ‘∆‐tAD’ mutant showed the same level of activity. When these constructs were tested in the more stringent chromatinized system described below, both were totally inactive (not shown). Western analysis showed very similar expression levels of the shortened mutants (Fig. 1D). We conclude that the LxxLL motif is not by itself sufficient to generate a functional tAD in c‐Myb.

### Strategy for mutagenesis of c‐Myb tAD

Since neither a pure acidic cluster of amino acids nor an isolated SLM is sufficient to generate a functional tAD in c‐Myb, we performed a more systematic analysis of key residues using a mutant scanning approach.

In addition to two previously studied mutants, the VNIV[267–270]ANAA (here abbreviated VNIV) [53] and M303V [54], we changed systematically the following residues:

1. Negatively charged residues were mutated to alanine in the following mutants
  - DED[286–288]AAA abbreviated DED
  - EKE[290–292]AKA abbreviated EKE
  - ELE[297–299]ALA abbreviated ELE
  - ENE[306–308]ANA abbreviated ENE
2. Positively charged residues were mutated to alanine in the following mutants
  - KEKRIK[291–296]AEAAIA abbreviated KEKRIK
  - RHY[282–284]AAY abbreviated RHY
3. Hydrophobic residues were mutated to alanine in the following mutants
  - LELLL[298–302]AELAA abbreviated LxxLL
  - Y284A
  - VL[314–315]AA abbreviated VL
  - WHSTT[327–331]AASAA abbreviated WHSTT

Acidic residues were targeted not only because of the classical ‘acid blob’ hypothesis, but also to address the relevance of the SLM‐exposure model of Staller *et al*. [5]. The basic residues were also targeted since they contribute to the charge and polarity of the region. The group of hydrophobic replacements, including the VNIV mutant, was expected to potentially affect small linear motifs (SLM) including the LxxLL motif referred to above. We have previously reported the VNIV mutant to affect binding to SUMO [53]. The WHSTT has not been described to have any function in c‐Myb, but the sequence resembles the motif WHTLF recently reported to be critical for interaction between androgen receptor and TFIIF [55].

All the mutants were generated on an EcoRI‐BglII fragment of the c‐Myb cDNA before the mutants were transferred to three c‐Myb expression vectors allowing tAD activity to be monitored in three different assay systems as described below.

### Analysis of tAD mutants in a standard effector–reporter assay

**Fig. 2:**
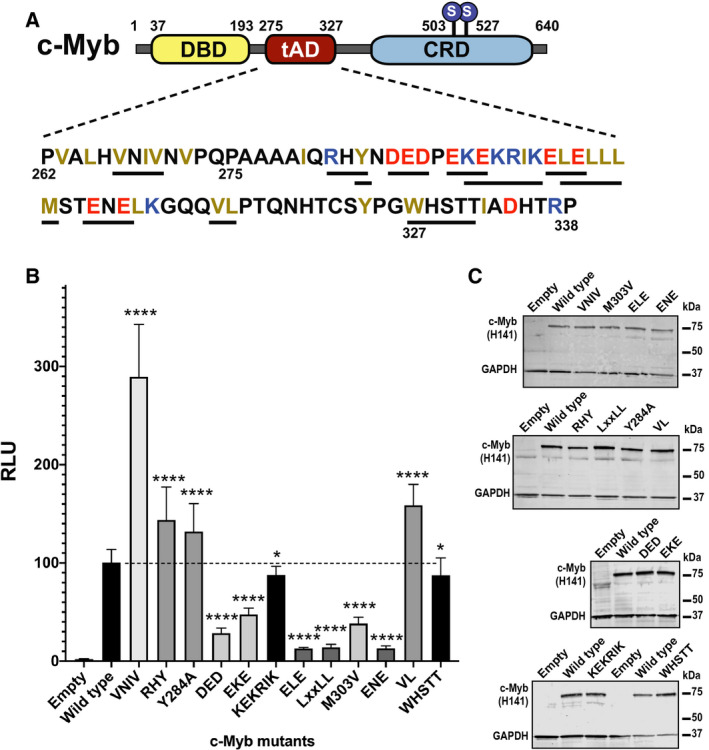
Mutational analysis of the tAD of c‐Myb. (A) Schematic presentation of human c‐Myb and the amino acid sequence of its wild‐type tAD subjected to mutational analysis with mutated residues underlined. (B) CV‐1 cells (0.2 × 10^5^ cells per well in 24‐well plates) were transfected with a Myb‐responsive pGL4b‐3xMRE(GG)‐myc reporter plasmid (0.2 μg) and plasmids encoding full‐length c‐Myb with indicated mutations (0.2 μg). The results are presented as relative luciferase units (RLU) with wild‐type set to 100. The results represent the mean RLU ± SD of at least three independent assays performed in triplicates. Significance was evaluated by unpaired, two‐tailed *t*‐tests on the selected mutant versus the wild‐type reference and indicated with *P*‐values (*P < 0.05; **P < 0.01; ***P < 0.001; ****P < 0.0001; ns, *P* > 0.05). (C) The western blots were analysed using anti‐c‐Myb (H141) and anti‐GAPDH primary antibodies.

Having generated this panel of mutants, we analysed how the mutations affected the transcriptional activity of c‐Myb in various reporter assays. In the first standard effector–reporter system, we used as effector an expression plasmid (pCIneoB‐hcM‐2KR) encoding an active version of c‐Myb not subject to SUMO‐mediated repression [28, 30] (c‐Myb‐2KR harbouring two mutations: K503R and K527R), into which all the tAD mutants were incorporated (Fig. 2A). A reporter construct, containing a synthetic c‐Myb‐responsive element (3xMRE) upstream of the luciferase gene, was transiently cotransfected with each of these c‐Myb expression plasmids in CV‐1 cells. Luciferase activities were measured 24 h after transfection. As shown in Fig. 2B, we found strong negative effects of removing acidic residues. All acidic mutants caused reduced transactivation.

However, we saw no clear correlation with charge, rather with position where ELE and ENE had stronger effect than the DED and EKE mutants. The ELE and ENE were the acidic mutants located closest to the LxxLL motif. These two had equally strong effect as eliminating the LxxLL motif itself. Mutating basic residues had only a marginally significant effect (*P* = 0.017).

In the SLM group, we observed a strong negative effect of mutating the LxxLL motif itself, consistent with its importance as a KIX domain interaction motif [39, 44, 45, 46, 47]. In line with previous observations, the VNIV mutant was threefold more active than the wild‐type. This SLM therefore appears to be involved in a repressive function, as previously reported [53]. The WHSTT motif does not seem to be very important for the activity of c‐Myb (*P* = 0.038) and therefore is probably not playing the same role as reported for androgen receptor [55].

There was a slight increase in activity by three mutants at some distance from the central LxxLL, namely RHY and Y284A on the N‐terminal side and VL on the C‐terminal side of the tAD, suggesting a slight restrictive effect of these residues. In the standard transfection assay, even the most affected mutants showed some residual activity above background. Western blot analysis showed no major effects on the steady‐state levels of the mutant c‐Myb proteins and none that could explain the most striking activity effects (Fig. 2C).

### Analysis of tAD mutants in a chromatinized assay

**Fig. 3:**
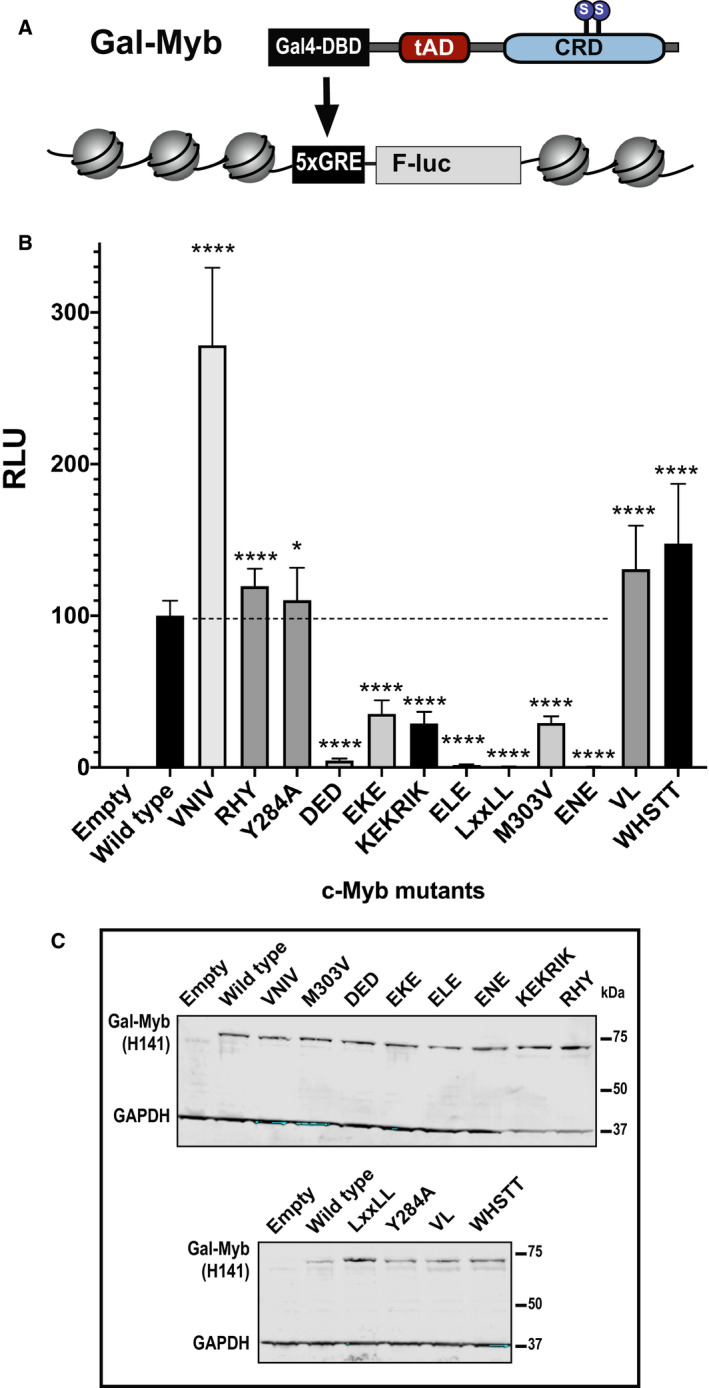
Mutational analysis of the tAD of c‐Myb using a chromatinized, embedded reporter. (A) Schematic presentation of the integrated reporter gene and the expressed Gal4p‐c‐Myb fusion protein harbouring the same mutations as shown in the Fig. 1 legend. (B) The HEK‐293‐derived reporter cell line, HEK293‐c1, was transfected with plasmids encoding the Gal4p‐c‐Myb fusion protein with indicated mutations (0.1 μg) and 0.3 μg pCIneoB empty vector. The results are presented as relative luciferase units (RLU) with wild‐type set to 100. The results represent the mean RLU ± SD of at least three independent assays performed in triplicates. Significance was evaluated by unpaired, two‐tailed *t*‐tests on the selected mutant versus the wild‐type reference and indicated with *P*‐values (*P < 0.05; **P < 0.01; ***P < 0.001; ****P < 0.0001; ns, *P* > 0.05). (C) The western blots were analysed using anti‐c‐Myb (H141) and anti‐GAPDH primary antibodies.

To determine whether activation of a gene embedded in chromatin had the same sensitivity to tAD mutants as a plasmid‐born reporter gene, we next examined how the mutants behaved in a more stringent system where the luciferase reporter is integrated in the genome. We therefore used a HEK 293‐c1 reporter cell line harbouring a 5×GRE Gal4‐luciferase reporter as an integrated transgene, where the reporter is mimicking a fully chromatinized target gene [48]. To direct the c‐Myb mutants to this site, we replaced the DBD of c‐Myb with that of yeast Gal4p‐DBD (Fig. 3A). In this system, we therefore used as effector an expression plasmid encoding a fusion between Gal4p‐DBD and an active version of c‐Myb not subject to SUMO‐mediated repression (c‐Myb 2KR), into which all the tAD mutants were incorporated. Luciferase activities were measured 24 h after transfection. As shown in Fig. 3B, we found many of the same overall effects of the mutants as in the standard transfection assay, but with a much lower background in this system. We measured strong negative effects of removing acidic residues. All acidic mutants caused reduced transactivation. The mutants with strongest effect in the first system were close to background and therefore basically inactive in this system. One difference was the triple‐acidic mutant DED, which had lost significantly more activity in this system than in the first system. Again, the strongest inhibitory effects among the acidic mutants were observed for those closest to the LxxLL motif, where ELE and ENE were totally inactive. Another difference was that we in this more stringent system observed a significant effect also of mutating basic residues. The KEKRIK mutant showed here only 29% activity. For the SLM group, mutating the central LxxLL motif fully abolished tAD function.

The VNIV mutant behaved similarly as in the first system being threefold more active than the wild‐type. The other peripheral mutants, RHY and Y284A on the N‐terminal side and VL and WHSTT on the C‐terminal side, had quite modest derepressive effects. Western blot analysis showed very similar expression levels of the majority of the mutants (Fig. 3C). The exception was the LxxLL mutant, showing increased steady‐state level, but still being totally inactive. We conclude that the tAD composition required to activate a target gene packed in chromatin is similar to that needed for a more loosely packed target gene, but in general, the effects of tAD mutants are augmented in the latter case, consistent with a critical role of the LxxLL motif in recruiting the histone acetyl transferase p300 to the chromatin‐embedded target through its KIX domain interaction.

### Analysis of tAD mutants in a yeast assay

Finally, we asked how conserved the mechanism giving the c‐Myb tAD its activity is? Would the mutants affect tAD similarly when expressed in a mammalian and a yeast system? Several recent reports have used transactivation assays in yeast as a readout for both yeast and mammalian tADs with the implicit assumption that the transactivation mechanism is highly conserved.

**Fig. 4:**
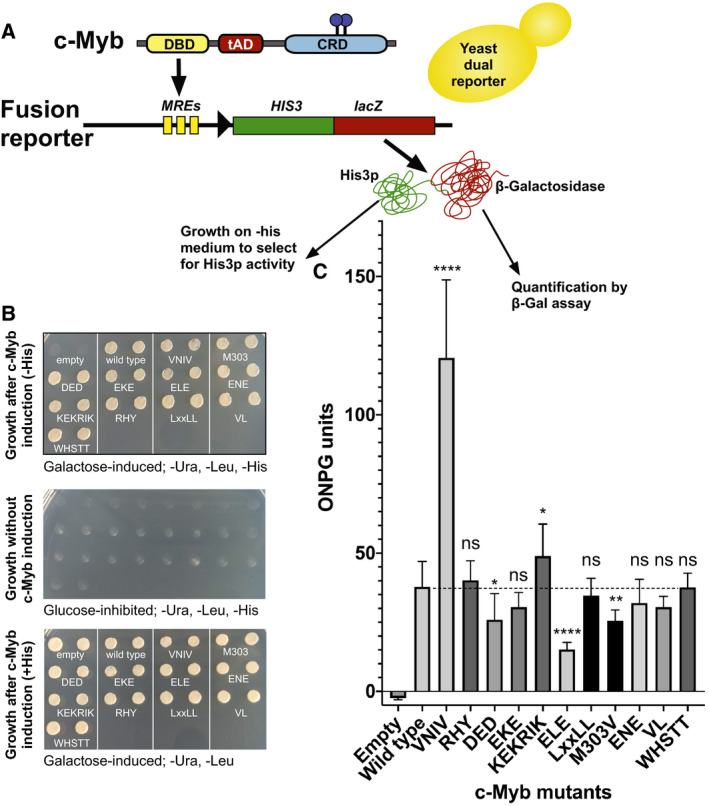
Mutational analysis of the tAD of c‐Myb in yeast. (A) Schematic presentation of the yeast effector–reporter system for monitoring the mutants of the tAD in c‐Myb. The reporter plasmid pGADT7‐HLfus‐3xGG contains a fusion of two reporter genes, *HIS3* and *lacZ*, under control of a c‐Myb‐responsive promoter (MRE). (B) Growth assays of spotted yeast clones transformed with the indicated mutant versions of c‐Myb and grown on the indicated conditions of selection and induction. (C) The β‐Gal activities for the indicated clones were measured under galactose‐induced conditions as described in Material and methods. The data in C are presented as mean β‐Gal values ± SD of three independent experiments, each carried out in triplicates. Significance was evaluated by unpaired, two‐tailed *t*‐tests on the selected mutant versus the wild‐type reference and indicated with *P*‐values (*P < 0.05; **P < 0.01; ***P < 0.001; ****P < 0.0001; ns, *P* > 0.05).

The alanine‐scanning c‐Myb mutants were first subcloned into a pYES2‐derived expression vector (pYES2‐hcM) allowing inducible expression of recombinant c‐Myb in yeast due to its pGAL1 promoter. As reporter, we used the dual‐reporter gene cassette from pYHLfus‐3xGG [50], where a fusion of *HIS3* and lacZ was driven by a synthetic Myb‐responsive promoter (Fig. 4A). This cassette was transferred from the original URA3 plasmid to the backbone of pGADT7‐AD to obtain independent plasmid selection markers (pGADT7‐HLfus‐3xGG). Finally, the *Saccharomyces cerevisiae* strain INVSc1 was transformed with the different expression plasmids encoding c‐Myb variants and the dual‐reporter plasmid. Three independent transformed colonies were picked and tested for reporter activation under different conditions of induction and selection.

As shown in Fig. 4B, the control with empty expression plasmid did not grow on a medium lacking histidine (upper panel), showing that growth under these conditions is dependent on c‐Myb‐mediated transactivation. The control was growing when supplemented with histidine (lower panel). Strikingly, all the c‐Myb‐expressing variants were growing on a medium lacking histidine. Any growth effects were at best marginal and not easy to estimate. Probably, the threshold for growth is reached already with low level of expression of the HIS3‐lacZ reporter. But it gives a first indication that the transactivation properties of c‐Myb in yeast differ from those of mammalian cells.

It appears that a study some time ago concluded that other parts of c‐Myb than the central tAD also contributes to tAD activity of c‐Myb in yeast [56]. If this is the case, such a second tAD would provide a constant contribution and have as effect an elevated background, probably sufficient for growth and thus explaining the growth effects observed here.

The β‐gal activity resulting from expression of the dual reporter is more suitable for quantification as illustrated in Fig. 4C. It would also allow effects of mutants to be seen even if the background should be elevated due to a second tAD function. Overall, we observed much less effects of the alanine‐scanning mutants here than in the mammalian systems, suggesting that despite the central c‐Myb tAD being active in yeast, it uses a mechanism that is more robust towards small alterations. Remarkably, mutation of the LxxLL motif had no effect in yeast, consistent with it being involved in interaction with a mammalian‐specific coactivator. Still, the c‐Myb tAD activates in yeast without an obvious SLM present. We notice that despite the existence of ‘fuzzy’ complexes, distinct specificities still operate for mammalian tADs.

Some of the mutations in acidic residues did not give significant effects. However, one of these mutants, ELE, had a clear negative effect, and another, DED, also reduced activation slightly. This pattern differed from the pattern seen in the mammalian assay systems. In yeast, it appears that overall charge may have an effect since neutralizing several basic residues in the KEKRIK mutant in fact made the tAD more active, in contrast to that in mammalian cells.

One of the striking similarities was the threefold increase in tAD activity of the VNIV mutant. This suggests either relief from a conserved interaction, such as SUMO/SMT3, or a more structural effect on the tAD itself. A similar behaviour was also observed for the M303V mutant.

## Discussion

The tADs of eukaryotic transcription factors have received renewed interest recently, and two new models for their mechanism of action have been proposed, the SLM‐exposure model [5] and the LLPS model [6]. In the light of these models, we revisited the tAD of the transcription factor c‐Myb and performed a mutational analysis that we designed and monitored in the light of these models. At the same time, we addressed the following questions.

- Which specific amino acid residues in c‐Myb tAD affect its transcriptional activity?
- Are acidic residues more critical for tAD function than basic residues?
- How does the SLM LxxLL affect transactivation function?
- Can we by mutagenesis find evidence for novel, not previously characterized SLMs in the tAD of c‐Myb?
- Has the wild‐type tAD sequence evolved to give a maximal activation effect or does some mutants increase, rather than decrease, its activation potential?

The tAD of human c‐Myb is acidic, and our mutational analysis shows that several acidic residues are critical for the activity of the tAD in mammalian cells, in particular in the system with a chromatinized reporter. In the latter system, mutating two or three of the centrally localized acidic residues totally abolishes transactivation. It is not a simple matter of charge since the KEKRIK mutation, removing four positive charges had only a minor effect in the first system and retained about 30% activity in the more sensitive system with a chromatinized reporter. Still, the acidic blob hypothesis is too simplistic to describe the c‐Myb tAD since mutating the hydrophobic SLM LxxLL is causing as severe drop in activity as the removal of acidic residues. The latter SLM is known to mediate interaction between c‐Myb tAD and the KIX domain of p300 and CBP [39, 44, 45, 46, 47], and our data suggest that this interaction is essential for the activity of the c‐Myb tAD in mammalian cells. We are left with a picture of two key elements in the dissected tAD. Both acidic residues and the central SLM are involved in creating an active tAD in c‐Myb. These also seem to cooperate locally since we see the most severe effects of altering the acidic residues closest to the LxxLL motif. This picture fits perfectly with the SLM‐exposure model of Staller *et al*. [5] proposing that both the SLM and the acidic residues cooperate, the latter by keeping the hydrophobic SLM exposed.

How does our observations relate to the recent LLPS model seeing tADs as domains inducing liquid–liquid phase separation? We have not directly tested our mutants for their ability to induce phase separation. But there are two arguments against a pure LLPS effect. The first argument relates to the type of mutants that apparently is needed to abolish LLPS‐forming properties. When LLPS mutants have been introduced in recent studies, they have been quite extensive, such as mutating all serines to alanine in the serine‐rich IDR of MED1 [23], or the change of a total of 17 acidic residues in the tAD of OCT4 [6], or changing either 12 or all 37 tyrosines in the prion‐like domain of the Ewing sarcoma fusion protein EWS‐FLI1 [57]. In several other studies, deletions of entire regions with intrinsically disordered properties have been studied, such as truncations of larger parts of the CTD in RNA polymerase II [21, 22], deletion of the 70 residue large histidine‐rich domain of cyclin T1 that promotes the hyperphosphorylation of the CTD and stimulates transcription through CDK9 [25], or deletions of histone tails [26]. Since LLPS is a biophysical phenomenon arising from a multitude of weak interactions, it is reasonable that major changes have to be introduced to alter these properties. It is therefore not very probable in our case to expect that changing two or three amino acid residues should be sufficient to fully abolish a tAD function if this function was solely a LLPS phenomenon. The second argument supporting the same conclusion is our yeast observations. A purely biophysical phenomenon should give a very similar outcome in mammalian and in yeast cells. This was not what we observed.

It should be noticed that the two models, the SLM‐exposure and the LLPS model, are not mutually exclusive. On the contrary, the LLPS model with its focus on multiple low‐affinity interactions clearly operates alongside the ability of transcription factors to form high‐affinity interactions with DNA and with coactivators [6].

Therefore, it is reasonable to assume a combination of the SLM‐exposure model and the LLPS model. This would imply a stepwise tAD action starting with a nucleation event where a specific interaction occurs between a SLM and a coactivator, the SLM being exposed and accessible due to its acidic environment, and a subsequent step where a LLPS process ensues due to the crowding that the nucleation initiated. It is also possible that it is the latter, less specific LLPS feature that operates in yeast providing sufficient clustering to initiate gene activation in that system. If this is so, one had to assume that the threshold for LLPS formation is lower in yeast than in mammalian cells or that our yeast expression plasmid leads to higher concentration of c‐Myb in yeast than our mammalian expression plasmid does in mammalian cells. In this way, one might explain why the mutants had less effect in the yeast system than in mammalian cells and that the mutated tADs remained active in yeast.

Two recent reports with impressive large‐scale analysis of tADs have used yeast as the basis for functional analysis of a large number of mutants in order to deduce features determining why a tAD operates as a tAD [5, 50]. The present study, where the same mutants have been analysed in both mammalian and yeast reporter assays, indicates that important features of a mammalian tAD can be missed when assayed in yeast. If we had based our analysis solely on the yeast assay, we would have concluded that the c‐Myb tAD is quite robust towards changes in two or three amino acid residues. The important role of both the LxxLL motif and the key importance of the acid residues close to that motif would have escaped our detection. The report of a second tAD function in c‐Myb, only seen when tested in yeast [56], is another example of divergent behaviour of tADs. This as a word of caution towards assuming that tAD function is so conserved that it can be investigated in remote heterologous systems. In this regard, it may be different from the DBDs, which will be functional as long as its interaction partner, a specific DNA sequence, is present. The interaction partners of a mammalian tAD are expected to have diverged substantially from yeast to mammalian organisms. Therefore, the effect of key residues in tAD may easily be missed in yeast.

The tAD of c‐Myb has to our knowledge not previously been subjected to this kind of detailed mutational analysis. In early days, its most important feature was regarded to be its acidic nature [58]. Later, the importance of its LxxLL motif came into focus [39, 44, 45, 46, 47], but their relative importance has not been compared. We found that both features were key elements in a functional tAD consistent with the SLM‐exposure model [5]. This is also emphasizing the key role that the tAD‐p300 interaction plays in the function of c‐Myb. Our observation of the strongest effects of the tAD mutants on the activation of a chromatin‐embedded reporter also makes sense for the recruitment of a histone modifier. Its importance for c‐Myb function is also underlined by the fact that this interaction has been used as a drug target for inhibiting c‐Myb function [59].

We did not find evidence for novel, not previously characterized SLMs in the tAD of c‐Myb. The effects we saw by mutating other hydrophobic residues were quite small and not supporting a key role in tAD function. The only exception was the already‐described VNIV motif, which we previously provided evidence being a SUMO‐interaction motif [53]. This also illustrates that the wild‐type tAD sequence has not evolved to give a maximal activation effect, since it is restricted by the effect of this repressive SLM.

## Conclusions

New model for tADs of eukaryotic transcription factors has recently been proposed, the SLM‐exposure model [5] and the LLPS model [6]. In this work, we performed a mutational analysis of the tAD of the transcription factor c‐Myb, which was interpreted in the light of these models. In mammalian cells, we observed a pattern of effects fully consistent with the SLM‐exposure model with strong effects of both acidic replacements and SLM mutation. The same mutants tested in a yeast system gave a quite different pattern, more as would be expected from a LLPS model. A two‐step hypothesis for transactivation, combining both models, is proposed.

## Conflict of interest

The authors declare no conflict of interest.

## Author contributions

GN, JOS and P‐DU shared the mutagenesis and plasmid design work and contributed to the writing and literature review. GN was responsible for all the experiments with first transfection system. P‐DU was responsible for all work with the chromatinized reporter system. JOS designed plasmids and executed all yeast work. ML supervised and assisted experimentally throughout the work. OSG was responsible for the overall experimental design, for supervision and for manuscript writing.

## Data Availability

The data sets used and/or analysed during the current study are available from the corresponding author upon reasonable request.
